# Hemorrhagic cerebral venous infarction after vein injury during intraoperative lesion resection: incidence, hemorrhagic stages, risk factors and prognosis

**DOI:** 10.3389/fneur.2024.1371184

**Published:** 2024-04-04

**Authors:** Yingxi Wu, Qilong Tian, Shoujie Wang, Kailu Li, Dayun Feng, Qing Cai

**Affiliations:** Department of Neurosurgery, Tangdu Hospital, Air Force Medical University, Xi'an, Shanxi, China

**Keywords:** cerebral venous infarction (CVI), hemorrhage, vein injury, prognosis, stage

## Abstract

**Objective:**

Cerebral venous infarction (CVI) after vein injury during intraoperative lesion resection is associated with intracranial hemorrhage. We conducted this study to identify the incidence, clinical and imaging features, and prognosis of hemorrhage CVI.

**Methods:**

We performed a retrospective analysis of patients with confirmed CVI after vein injury who underwent craniotomy in our hospital. Postoperative clinical symptoms were observed, and imaging features were compared between patients with and without intracranial hemorrhages through CT examination. Variables were analyzed using univariate and multivariate regression analyses.

**Results:**

Among 2,767 patients who underwent craniotomy, 93 cases of injured veins were identified intraoperatively. Hemorrhagic CVI was found in 38% (35/93). Multivariate analysis revealed that midline approach, meningioma, postoperative seizures, disorders of consciousness and interval in hours < 72 h were identified as predictors of hemorrhagic CVI. After 3 months of follow-up, the prognosis was poor in 15 cases (16%, 15/93), including death (two cases), vegetative survival (four cases), and severe disability (nine cases).

**Conclusions:**

Hemorrhagic CVI, as a critical complication after venous injury, can have disastrous consequences. Do not injure known veins intraoperatively. In case of injury, requisite remedial measures should be adopted during and after surgery.

## Introduction

Cerebral venous infarction (CVI) after vein injury during intraoperative lesion resection is a complication that cannot be ignored. Hemorrhagic CVI is an important cause of death and disability in patients postoperatively (1). On the basis of a literature review, the incidence of venous injury can range from 2.6 to 30%, and the incidence of CVI after injury is 0.15–13% (2). However, apart from multiple case reports and small case series, the literature on hemorrhagic venous infarction in the setting of intraoperative vein injury has been scarce.

When the drainage vein is injured, the vein lumen at the injured site is narrowed or blocked, the blood flow velocity upstream of the injured vein slows, and the venous pressure of the secondary venules and capillaries increases. To alleviate the increase in intracranial pressure, the intracranial veins are compensated for by expanding the diameter of the venous vessels, recanalizing the original closed pathway or neovascularization (3). If the compensation is adequate, there might be no obvious change in the dominant area of the injured vein. If the collateral compensation of the veins is limited, blood flow in the vein injury area decreases, and congestion in the lumen, tissue ischemia and hypoxia, an increase in capillary permeability and destruction of the blood brain barrier gradually leads to edema of the local brain parenchyma, cerebral infarction and cerebral parenchyma hemorrhage (4). Research showed (5) that 11% (8/73) of patients with secondary venous thrombosis after cerebral trauma developed cerebral hemorrhage. Several studies (6–8) have emphasized that cerebral venous thrombosis (CVT) is associated with intracranial hemorrhage in up to 39–40% of patients. In a mouse CVI model after cortical vein injury, the hemorrhagic CVI rate of our research group reached 60–80%, and imaging and histology showed subcortical or cerebral parenchyma hemorrhage (9).

Whether CVI or hemorrhagic CVI occurs after venous injury depends on the quantity of anastomotic branches in the dominant area of the injured vein. Hemorrhagic CVI is the most serious clinical manifestation of venous decompensation. Therefore, we systematically reviewed 93 patients with CVI after vein injury during surgery, analyzed the incidence of and risk factors for hemorrhagic CVI, and identified the veins responsible for injury to provide a theoretical basis for reducing hemorrhagic CVI after vein injury.

## Methods

### Study population

A retrospective study was performed on 2,767 patients who underwent intracranial lesion resection in the Department of Neurosurgery, Tangdu Hospital of the Air Force Medical University (Xi'an, China), from January 2011 to December 2021. Venous vessels were definitely injured intraoperatively in 93 patients, and it was assumed that venous-related edema, infarction and hemorrhage occurred postoperatively. We collected patient data from electronic medical records and radiology systems. The criteria for exclusion were as follows: (1) emergency surgery for brain trauma, cerebral hemorrhage and cerebral infarction; (2) surgery related to vascular diseases, such as aneurysm, arteriovenous malformation, etc.; and (3) venous edema, infarction and hemorrhage caused by injury or stenosis of the intracranial venous sinus. All study procedures were approved by the ethics committee of Tangdu and followed the guidelines of the Helsinki Declaration. As per the terms of this ethical approval, the necessity for individual patient consent was waived.

### Variables and data collection

**Figure 1:**
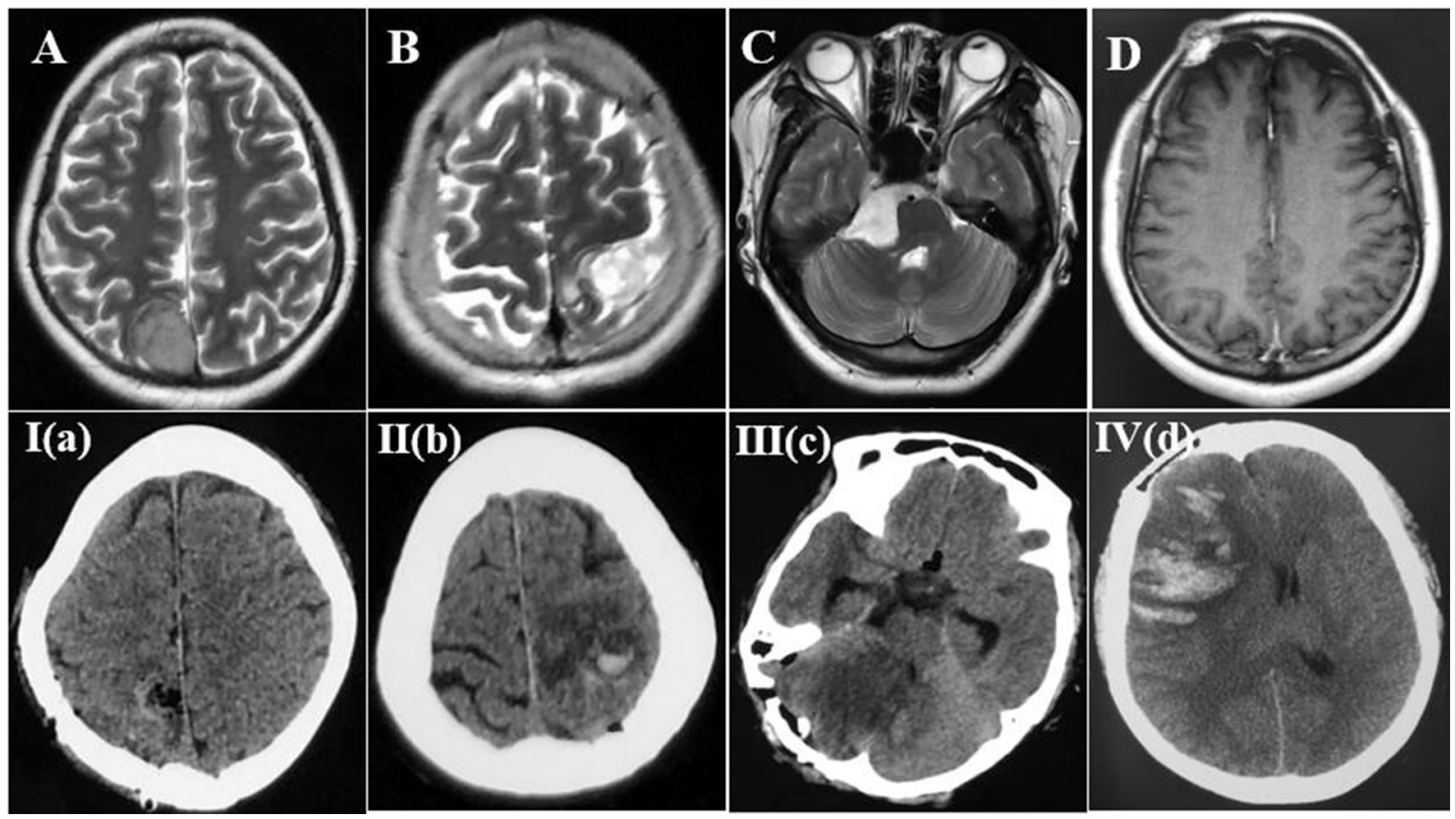
Imaging of CVI after venous injury. **(A)** Right parietal parasagittal meningioma. (a) Injure part of the cortical system, no change in brain tissue after tumor resection (I). **(B)** left parietal convexity meningioma. (b) Injury of the postcentral vein, local edema, infarction and minor hemorrhage of brain tissue after tumor resection (II). **(C)** Epidermoid cyst in right cerebellopontine angle. (c) Injury of superior petrosal vein, severe edema and infarction of cerebellar tissue, compression of fourth ventricle after tumor resection (III). **(D)** pathological confirmation of inflammatory granuloma. (d) Injure middle frontal vein, cerebral hemorrhage, edema and infarction, obvious midline shift (IV).

All patient data were collected from our hospital electronic medical records. Follow-up data (at least 3 months) were obtained via telephone interviews. Clinical data, such as age, sex, and postoperative clinical manifestations (no symptoms, headache, focal neurological deficits, seizure and coma), were retrieved. Tumor size was determined based on the maximum axial diameter on gadolinium-enhanced T1-weighted and FLAIR MRI sequences. Pathological grading was based on the 2016 WHO criteria. Surgical approaches included the midline, frontotemporal, subfrontal, subtemporal, suboccipital, transcortical, sigmoid sinus and far lateral approaches, etc. Whether to undergo reoperation for edema, infarction, and bleeding after venous injury should be evaluated by two senior clinicians according to the patient's symptoms and imaging. The methods of craniotomy included hematoma evacuation and/or decompressive craniectomy. Disorder of consciousness was defined as drowsiness, lethargy and coma. The interval was defined as the time when the symptoms related to venous injury appeared postoperatively and was confirmed by head CT examination. We routinely performed cranial MRI and MRV examinations to determine the anatomical position of the veins according to sulcus, gyrus, and lesions preoperatively, and judged the consistency based on the observed veins around the lesion intraoperatively and the veins in preoperative MRV. CVI (postoperative CT) was defined as a new or a larger low-density area around the lesion resection. Hemorrhagic CVI was defined as hemorrhage around the low-density area in the brain parenchyma with a mass effect postoperatively. A new or a larger low-density area was located in the scope of the injured venous drainage. No density change in the area of injured venous drainage was considered asymptomatic CVI. Based on the severity of symptoms after venous injury, imaging findings were divided into four stages referring to the previous stages of venous sinus occlusion (10) (I: no change, II: mild edema III: severe edema/infarction, with/without hemorrhage, IV: massive edema/infarction and hemorrhage; Figure 1). We regarded a GOS score of 4–5 as a good prognosis and a GOS score of 1–3 as a poor prognosis.

### Statistical analysis

The results are presented as counts (percentage). Pearson's chi-square test or Fisher's exact was used to compare differences between the distribution of involved venous vessels in the hemorrhagic and non-hemorrhagic groups. The variables that were associated with hemorrhagic CVI were identified in univariate analysis (*p* < 0.10) and then entered into multivariate logistic regression. All statistical analyses were performed using SPSS software (version 24, IBM Corp.), and *p* < 0.05 was considered statistically significant.

## Results

Among 2,767 patients who underwent craniotomy, 93 cases of injured veins were identified intraoperatively. Postoperative venous infarction was confirmed by CT. The incidence of vein injury-related edema, infarction and hemorrhage was 3.3% (93/2,767), and the incidence of hemorrhagic CVI was 38% (35/93). There were cases of asymptomatic CVI (26 cases), mild symptomatic CVI (32 cases), and severe symptomatic CVI (hemorrhagic 35 cases). The types of lesions with injured veins included meningiomas (47 cases), gliomas (23 cases), acoustic neuromas (14 cases), trigeminal neuralgia (five cases), facial spasm (two cases), inflammatory granuloma (one case), and chronic subdural hematoma with calcification (one case). In cases of hemorrhagic CVI, there were meningiomas (23 cases), gliomas (six cases), acoustic neuromas (three cases), facial spasm (one case), inflammatory granuloma (one case), and chronic subdural hematoma with calcification (one case).

**Figure 2:**
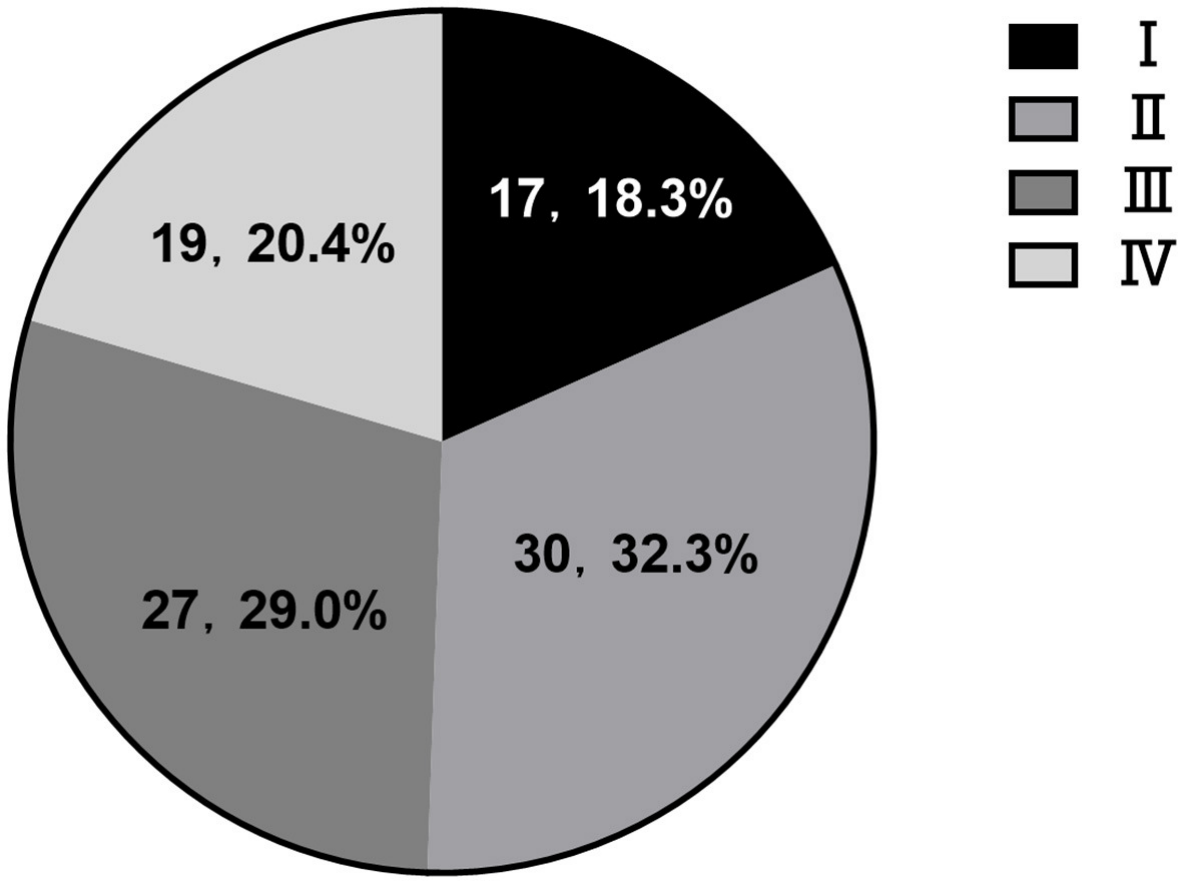
Proportion of CVI in each stage.

The proportion of CVI in each stage was 18.3% (I), 32.3% (II), 29.0% (III), and 20.4% (IV), respectively. Stage III and IV cases (83%, 38/46) were mostly hemorrhagic CVI, which were accompanied by obvious mass effect and hemorrhage. The majority of patients underwent surgical treatment. The cases with poor prognosis were also distributed in stage III and IV. Among 93 patients with venous injury, 61 cases received conservative treatment and had a good prognosis, accounting for 95.1% (58/61). Surgical treatment had a good prognosis in 32 cases, accounting for 62.5% (20/32). Conservative (two cases) and craniotomy (four cases) had severe poor prognosis (GOS1-2), mainly in cases of injury to the internal/great cerebral vein, central vein and Labbé vein. Poor prognosis (GOS3) was focused on the middle frontal vein (two cases) and posterior frontal vein (three cases) (Figure 2; Table 1).

**Table 1:** The distribution of prognosis in conservative and surgical patients after venous injury.

**CVI**	**Prognosis**				
	**GOS1**	**GOS2**	**GOS3**	**GOS4**	**GOS5**
Conservative (61)	–	2	1	13	45
Surgery (32)	2	2	8	9	11

### Risk factors related to hemorrhagic CVI

#### Univariate analysis

Patients who underwent the midline approach significantly more often exhibited hemorrhagic CVI than those who underwent other approaches (54.3 vs. 45.7%, *p* = 0.045, OR 2.60, 95% CI 1.09–6.34). Meningioma was significantly more often associated with hemorrhagic CVI than other lesion types (65.7 vs. 34.3%, *p* = 0.039, OR 2.67, 95% CI 1.13–6.60). Patients suffering from seizures significantly more often exhibited hemorrhagic CVI than did those without seizures (68.6 vs. 31.4%, *p* = 0.005, OR 3.76, 95% CI 1.56–9.55). Patients with disorders of consciousness suffered from hemorrhagic CVI significantly more often than those without disorders of consciousness (77.1 vs. 22.9%, *p* < 0.001, OR 6.21, 95% CI 2.45–17.2). Patients with an interval of < 72 h significantly more often suffered from hemorrhagic CVI than did those without an interval of ≥72 h (65.7 vs. 34.3%, *p* < 0.001, OR 5.85, 95% CI 2.37–15.3) (Tables 2, 3).

**Table 2:** Baseline characteristics of the patients with CVI and hemorrhagic CVI.

**Variable**	**Total CVI**	**Non-hemorrhagic CVI**	**Hemorrhagic CVI**	***p*-value**
	**(*****n*** = **93)**	**(*****n*** = **58)**	**(*****n*** = **35)**	
**Sex**				0.931
Female	55 (59.1%)	35 (60.3%)	20 (57.1%)	
Male	38 (40.9%)	23 (39.7%)	15 (42.9%)	
**Age**				0.728
<60	46 (49.5%)	30 (51.7%)	16 (45.7%)	
≥60	47 (50.5%)	28 (48.3%)	19 (54.3%)	
**Lesion size (mm)** ^**#**^				0.669
<30	42 (47.2%)	24 (44.4%)	18 (51.4%)	
≥30	47 (52.8%)	30 (55.6%)	17 (48.6%)	
**WHO grade III-IV** ^**&**^				0.241
No	61 (68.5%)	34 (63.0%)	27 (77.1%)	
Yes	28 (31.5%)	20 (37.0%)	8 (22.9%)	
**Surgical approach**				0.045
Others	56 (60.2%)	40 (69.0%)	16 (45.7%)	
Midline approach	37 (39.8%)	18 (31.0%)	19 (54.3%)	
**Diagnosis**				0.039
Others	46 (49.5%)	34 (58.6%)	12 (34.3%)	
Meningioma	47 (50.5%)	24 (41.4%)	23 (65.7%)	
**Headache**				0.461
No	14 (15.1%)	7 (12.1%)	7 (20.0%)	
Yes	79 (84.9%)	51 (87.9%)	28 (80.0%)	
**Seizures**				0.005
No	48 (51.6%)	37 (63.8%)	11 (31.4%)	
Yes	45 (48.4%)	21 (36.2%)	24 (68.6%)	
**Focal neurological signs**				0.290
No	14 (15.1%)	11 (19.0%)	3 (8.57%)	
Yes	79 (84.9%)	47 (81.0%)	32 (91.4%)	
**Disorder of consciousness**				<0.001
No	46 (49.5%)	38 (65.5%)	8 (22.9%)	
Yes	47 (50.5%)	20 (34.5%)	27 (77.1%)	
**Interval in hours**				<0.001
≥72	56 (60.2%)	44 (75.9%)	12 (34.3%)	
<72	37 (39.8%)	14 (24.1%)	23 (65.7%)	

^**#**^In total: 89 cases.

^&^In total: 89 cases; WHO grade II–III (meningioma) is equivalent to WHO grade III–IV (other tumors).

**Table 3:** Clinical risk factors for prediction of hemorrhagic CVI.

**Variable**	**Univariable analysis**		**Multivariable analysis**	
	**OR (95%CI)**	***P***	**OR (95%CI)**	***P***
Sex (female vs male)	1.14 (0.48–2.69)	0.931		
Age (years) (<60 vs. ≥60)	1.27 (0.54–2.99)	0.728		
Lesion size (mm) (<30 vs. ≥30)	0.76 (0.32–1.79)	0.669		
WHO grade III–IV (no vs. yes)	1.95 (0.76–5.42)	0.241		
Surgical approach (others vs. midline approach)	2.60 (1.09–6.34)	0.045	3.27(1.07–10.03)	0.038
Diagnosis (others vs. meningioma)	2.67 (1.13–6.60)	0.039	3.18(1.01–10.00)	0.048
Headache (No vs. yes)	0.55 (0.17–1.81)	0.461		
Seizures (No vs. yes)	3.76 (1.56–9.55)	0.005	4.25(1.38–13.05)	0.012
Focal neurological signs (no vs. yes)	2.40 (0.67–11.8)	0.290		
Disorder of consciousness (no vs. yes)	6.21 (2.45–17.2)	<0.001	5.22(1.67–16.4)	0.004
Interval in hours (≥72 vs. <72)	5.85 (2.37–15.3)	<0.001	6.53(2.06–20.7)	0.001

#### Multivariate analysis

We performed a multivariate logistic regression analysis to identify potential predictors of hemorrhagic CVI in postoperative patients with intracranial lesions. The presence of a midline approach (*p* = 0.038, OR 3.27, 95% CI 1.07–10.03), meningioma (*p* = 0.048, OR 3.18, 95% CI 1.01–10.00), seizures (*p* = 0.012, OR 4.25, 95% CI 1.38–13.05), disorders of consciousness (*p* = 0.004, OR 5.22, 95% CI 1.67–16.4), and an interval < 72 h (*p* = 0.001, OR 6.53, 95% CI 2.06–20.7) were identified as significant predictors of postoperative hemorrhagic CVI (Table 3).

### Prognosis of venous infarction staging after responsible vessel injury

**Figure 3:**
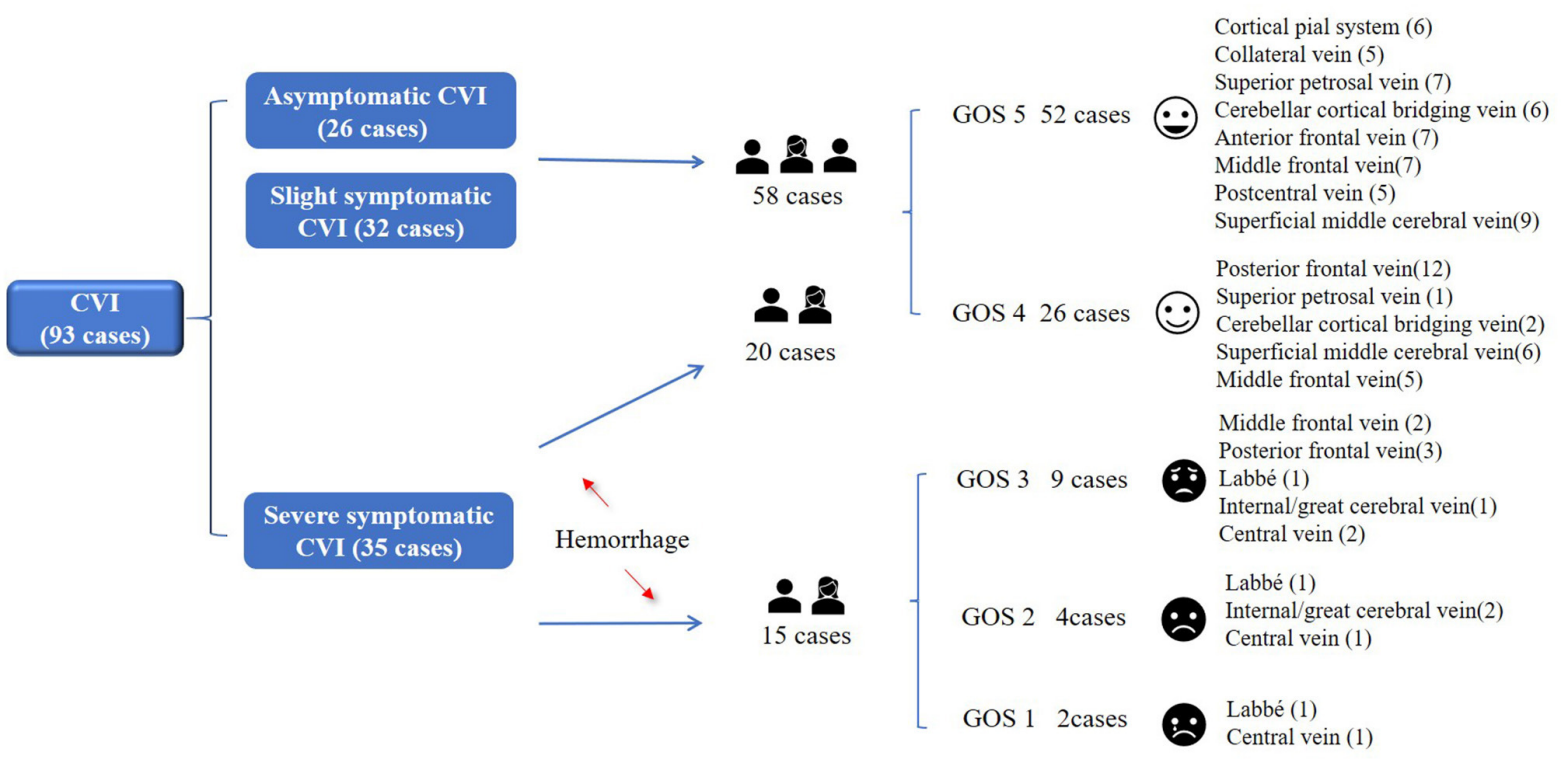
Prognosis of CVI after injury to various veins. GOS, Glasgow Outcome Scale.

The GOS score was closely related to the injured vein (Figure 3; Supplementary Table). Patients after CVI were discharged following conservative or surgical treatment. The patients had been followed up for more than 3 months.

The prognosis was good in 78 cases (84%, 78/93). The responsible vessel included cortical pial system (six cases), collateral vein (five cases), superior petrosal vein (eight cases), cerebellar cortical bridging vein (eight cases), anterior frontal vein (seven cases), postcentral vein (five case), superficial middle cerebral vein (15 cases), middle frontal vein (12 cases) and posterior frontal vein (13 cases). The prognosis was poor in 15 cases (16%, 15/93). The responsible vessel included middle frontal vein (two cases), posterior frontal vein (three cases), Labbé (three cases), central vein (four cases) and internal/great cerebral vein (three cases). The clinical outcome was death (two cases, coma-associated pneumonia), vegetative survival (four cases) and severe disability (nine cases).

The hemorrhagic cases with more injured veins and obvious symptoms were middle frontal vein (14 cases) and posterior frontal vein (15 cases). These were poor prognosis rates of 14% (2/14) and 20% (3/15), respectively. Once Labbé, central vein and internal/great cerebral vein were injured, it would inevitably cause CVI or hemorrhagic CVI, leading to irreversible severe neurological dysfunction and even death.

## Discussion

Cerebral venous infarction refers to the infarction focus formed after cerebral ischemia, hypoxia, edema, necrosis and hemorrhage due to venous factors, and it is mostly caused by cerebral drainage veins or venous sinus thrombosis, infection, trauma, tumors, etc. (11, 12). Surgery-related CVI is caused by accidental vein injury or purposeful vein sacrifice during surgery. To better expose the focus, the venous vessels that can be electrocoagulated or the venous sinuses that can be ligated in the surgical approach are sacrificed, such as the frontal drainage vein and the corresponding anterior 1/3 of the sagittal sinus (13), the corresponding drainage vein in the posterior 1/3 sagittal sinus (14), and the superior petrosal vein in the cerebellopontine angle (15, 16). However, according to case reports (17), series reports (18) and systematic analyses of CVI (19–21), hemorrhagic CVI has the characteristics of short onset time, serious clinical manifestations and poor prognosis, which should be acted upon urgently (22).

### Incidence and risk factors of hemorrhagic venous cerebral infarction

The article clarified the incidence of hemorrhagic cerebral venous infarction (CVI) after venous injury postoperatively during surgery, and confirmed risk factors for hemorrhagic venous cerebral infarction. The incidence of hemorrhagic CVI was 38% (35/93), with poor outcomes in ~16% (15/93) of cases, similar to that with venous thrombosis hemorrhage (6–8, 23). Our research group reviewed the cases of meningioma resection in our hospital, combined them with the relevant literature to identify the risk factors for CVI (21), and further analyzed the risk factors for a large sample of patients with hemorrhagic CVI after intracranial lesion resection. There was a significant difference between hemorrhagic CVI and non-hemorrhagic CVI, and the difference was in the clinical symptoms and prognosis after surgery. The results showed that hemorrhagic CVI was mostly seen in meningiomas, especially superficial meningiomas, in which the postoperative CVI rate was as high as 5.5% (24). Similarly, to increase the surgical field of vision, lesion resection through the midline approach often sacrifices the bridging vein, increasing the probability of hemorrhage. Hemorrhagic symptoms occurred earlier than simple edema/infarction. The interval postoperatively was within 72 h, and the clinical manifestation was sudden seizure and coma, indicating brain function decompensation after acute hemorrhage.

### Prognosis of CVI after responsible venous injury

The prognosis of CVI patients was closely related to the responsible veins for injury. We analyzed the prognosis of CVI patients with natural venous injuries during intraoperative lesion resection. The imaging staging after venous injury reflected the severity of the patient's condition to a certain extent. The higher the grading, the more severe the condition, and the higher the proportion of poor prognosis. The results indicated that the injured central vein, vein of Labbé, middle frontal vein and posterior frontal vein were more likely to bleed. Most patients needed emergency craniotomy to remove the hematoma after bleeding, and the bone flap was removed for decompression if necessary (25). Timely intervention has a good prognosis, but injuries to the central vein and vein of Labbé result in irreversible neurological dysfunctions and even death (26). Internal/great cerebral vein injury is mainly caused by infarction, with minor hemorrhages but poor prognosis. The preservation of the SPV vein is a neurosurgical dilemma (16, 27). A literature review and experiences from large series have shown that obliterating the vein of SPV could be associated with negligible complications. However, the opposite view cannot be ignored in light of some series showing a complications rate of up to 30% (28–30). The cortical pial system is usually located among the arachnoid membrane, pia mater and cortex (31). It is inevitable to injure the venous system when removing lesion. Minor venous injury will not cause symptoms, and excessive venous system injury can lead to mild edema, infarction and minor bleeding without causing serious complications. The anterior frontal vein and posterior central veins dominate a small area of drainage, have no important functional areas, and generally do not show clinical symptoms after injury. Because of the rich collateral circulation of the superficial middle cerebral vein, some injuries will not cause obvious symptoms (32). Cerebellar cortical bridging veins are mostly seen in the retrosigmoid and supracerebellar infratentorial approach, but the risk of CVI is an unpredictable (33). When the cerebellum is pulled during the operation, the bridging veins that enter the transverse sinus/sigmoid sinus will be injured. Cerebellar edema is often seen, and cerebellar parenchyma hemorrhage is occasionally seen. The prognosis is good through conservative treatment or removal of the hematoma.

## Conclusions

Hemorrhagic CVI is the most serious complication after venous injury, and the veins through the surgical channel or the perilesional veins should be protected by various means because of the uncertainty of collateral compensation and variability of the vein. Infarction and hemorrhage after vein injury cannot be completely avoided even through comprehensive evaluation. Critical venous (Labbé or central vein) injury requires intraoperative venous reconstruction. If the consequences of venous injury or venous anastomosis failure cannot be predicted, the clinical manifestations of patients should be closely observed, a timely CT examination should be conducted postoperatively, and early intervention (drugs and surgery) should be performed to prevent nerve dysfunction caused by venous injury.

## Data availability statement

The raw data supporting the conclusions of this article will be made available by the authors, without undue reservation.

## Ethics statement

The studies involving humans were approved by Ethics Committee of Tangdu Hospital. The studies were conducted in accordance with the local legislation and institutional requirements. The participants provided their written informed consent to participate in this study. Written informed consent was obtained from the individual(s) for the publication of any potentially identifiable images or data included in this article.

## Author contributions

YW: Conceptualization, Formal analysis, Writing – original draft. QT: Methodology, Writing – review & editing. SW: Investigation, Writing – original draft. KL: Investigation, Writing – review & editing. DF: Supervision, Writing – review & editing. QC: Writing – original draft, Writing – review & editing.
