# Supplementary material for: Hemorrhagic cerebral venous infarction after vein injury during intraoperative lesion resection: incidence, hemorrhagic stages, risk factors and prognosis
Source: Front Neurol. 2024 Apr 4;15:1371184. doi: 10.3389/fneur.2024.1371184 (PMC11034368; doi:10.3389/fneur.2024.1371184)
Supplement: Supplementary file 1 [file Table_1.DOCX]

|  | **Age/Sex** | **Diagnosis** | **Surgical approaches** | **Injured vein** | **Injured result** | **Prognosis**  **(GOS)** |
| --- | --- | --- | --- | --- | --- | --- |
| 1 | 56/F | Meningioma | midline | MFV | hemorrhage | 4 |
| 2 | 34/F | Acoustic neuromas | sigmoid sinus | SPV | no change | 5 |
| 3 | 38/M | Gliomas | transcortical | I/GCV | hemorrhage | 3 |
| 4 | 69/M | Meningioma | frontotemporal | AFV | infarction | 5 |
| 5 | 65/F | Meningioma | occipital | Collateral vein | no change | 5 |
| 6 | 55/M | Meningioma | frontal | CPS | no change | 5 |
| 7 | 46/F | Acoustic neuromas | sigmoid sinus | SPV | edema | 5 |
| 8 | 61/F | Meningioma | midline | MFV | hemorrhage | 4 |
| 9 | 54/M | Trigeminal neuralgia | sigmoid sinus | Collateral vein | no change | 5 |
| 10 | 38/F | Meningioma | midline | PFV | infarction | 4 |
| 11 | 67/M | Acoustic neuromas | sigmoid sinus | SPV | edema | 5 |
| 12 | 26/F | Gliomas | midline | AFV | infarction | 5 |
| 13 | 67/F | Meningioma | subtemporal | Labbé | hemorrhage | 1 |
| 14 | 69/M | Meningioma | midline | MFV | edema | 5 |
| 15 | 71/F | Meningioma | midline | MFV | infarction | 5 |
| 16 | 33/M | Gliomas | transcortical | AFV | no change | 5 |
| 17 | 39/F | Gliomas | transcortical | SMCV | hemorrhage | 4 |
| 18 | 71/M | Meningioma | midline | MFV | hemorrhage | 3 |
| 19 | 42/F | Grigeminal neuralgia | sigmoid sinus | SPV | edema | 5 |
| 20 | 46/F | Acoustic neuromas | far lateral | SPV | edema | 5 |
| 21 | 61/M | Meningioma | midline | MFV | hemorrhage | 3 |
| 22 | 57/F | Meningioma | temporal | SMCV | edema | 5 |
| 23 | 34/F | Gliomas | transcortical | I/GCV | hemorrhage | 2 |
| 24 | 68/F | Meningioma | midline | CPS | no change | 5 |
| 25 | 69/F | Meningioma | suboccipital | Postcentral vein | edema | 5 |
| 26 | 43/M | Meningioma | midline | MFV | edema | 5 |
| 27 | 77/M | Acoustic neuromas | sigmoid sinus | CCBV | edema | 5 |
| 28 | 54/F | Gliomas | midline | AFV | infarction | 3 |
| 29 | 64/F | Trigeminal neuralgia | sigmoid sinus | SPV | no change | 5 |
| 30 | 65/F | Meningioma | midline | MFV | infarction | 4 |
| 31 | 49/M | Gliomas | transcortical | AFV | edema | 5 |
| 32 | 48/F | Gliomas | midline | PFV | hemorrhage | 3 |
| 33 | 52/F | Meningioma | midline | Central vein | hemorrhage | 1 |
| 34 | 42/M | Gliomas | transcortical | AFV | edema | 5 |
| 35 | 51/F | Meningioma | transcortical | SMCV | hemorrhage | 4 |
| 36 | 55/M | Gliomas | midline | MFV | edema | 5 |
| 37 | 36/F | Meningioma | midline | MFV | edema | 5 |
| 38 | 43/M | Gliomas | transcortical | Collateral vein | no change | 5 |
| 39 | 64/F | Acoustic neuromas | sigmoid sinus | Collateral vein | no change | 5 |
| 40 | 66/M | Meningioma | midline | PFV | hemorrhage | 4 |
| 41 | 61/F | Meningioma | midline | MFV | hemorrhage | 4 |
| 42 | 58/M | Trigeminal neuralgia | sigmoid sinus | CPS | no change | 5 |
| 43 | 71/F | Meningioma | midline | PFV | hemorrhage | 3 |
| 44 | 76/M | Meningioma | transcortical | SMCV | infarction | 4 |
| 45 | 68/F | Gliomas | midline | PFV | hemorrhage | 4 |
| 46 | 56/M | Trigeminal neuralgia | sigmoid sinus | SPV | infarction | 5 |
| 47 | 61/M | Meningioma | midline | PFV | hemorrhage | 3 |
| 48 | 65/M | Meningioma | subtemporal | Labbé | hemorrhage | 2 |
| 49 | 67/F | Gliomas | midline | MFV | hemorrhage | 4 |
| 50 | 42/M | Gliomas | transcortical | SMCV | edema | 5 |
| 51 | 37/F | Acoustic neuromas | far lateral | CCBV | hemorrhage | 4 |
| 52 | 63/M | Meningioma | transcortical | SMCV | infarction | 4 |
| 53 | 59/F | Meningioma | midline | Central vein | hemorrhage | 2 |
| 54 | 54/F | Gliomas | transcortical | CPS | no change | 5 |
| 55 | 68/M | Gcoustic neuromas | sigmoid sinus | SPV | hemorrhage | 4 |
| 56 | 79/F | Meningioma | transcortical | I/GCV | hemorrhage | 2 |
| 57 | 64/M | Facial spasm | sigmoid sinus | CCBV | edema | 5 |
| 58 | 66/F | Meningioma | midline | MFV | edema | 5 |
| 59 | 48/F | Acoustic neuromas | sigmoid sinus | CCBV | infarction | 5 |
| 60 | 78/M | Meningioma | midline | PFV | hemorrhage | 4 |
| 61 | 36/F | Gliomas | transcortical | SMCV | edema | 5 |
| 62 | 37/M | Meningioma | midline | Central vein | hemorrhage | 3 |
| 63 | 58/F | Gliomas | transcortical | SMCV | edema | 5 |
| 64 | 65/M | Acoustic neuromas | sigmoid sinus | CCBV | infarction | 5 |
| 65 | 69/F | Meningioma | midline | PFV | hemorrhage | 4 |
| 66 | 70/F | CSH (calcification) | frontotemporal | MFV | hemorrhage | 4 |
| 67 | 57/F | Gliomas | frontotemporal | SMCV | edema | 5 |
| 68 | 54/F | Meningioma | midline | PFV | hemorrhage | 4 |
| 69 | 46/M | Meningioma | frontotemporal | SMCV | infarction | 4 |
| 70 | 58/F | Acoustic neuromas | sigmoid sinus | CCBV | no change | 5 |
| 71 | 63/M | Meningioma | midline | Central vein | hemorrhage | 3 |
| 72 | 35/F | Gliomas | transcortical | AFV | edema | 5 |
| 73 | 23/F | Meningioma | suboccipital | Postcentral vein | edema | 5 |
| 74 | 78/F | Meningioma | midline | PFV | hemorrhage | 4 |
| 75 | 64/M | Acoustic neuromas | sigmoid sinus | CPS | no change | 5 |
| 76 | 70/F | Meningioma | temporal | SMCV | hemorrhage | 4 |
| 77 | 52/F | Gliomas | transcortical | SMCV | infarction | 4 |
| 78 | 67/F | Meningioma | midline | PFV | infarction | 4 |
| 79 | 40/M | Gliomas | transcortical | Postcentral vein | edema | 5 |
| 80 | 60/F | Meningioma | temporal | PFV | hemorrhage | 4 |
| 81 | 17/F | Inflammatory granuloma | transcortical | SMCV | edema | 5 |
| 82 | 63/M | Acoustic neuromas | sigmoid sinus | CCBV | hemorrhage | 4 |
| 83 | 45/F | Meningioma | subtemporal | Labbé | hemorrhage | 3 |
| 84 | 78/M | Meningioma | midline | Postcentral vein | no change | 5 |
| 85 | 23/F | Gliomas | temporal | SMCV | edema | 5 |
| 86 | 63/M | Meningioma | frontal | SMCV | edema | 5 |
| 87 | 65/F | Facial spasm | sigmoid sinus | Collateral vein | no change | 5 |
| 88 | 74/F | Meningioma | midline | PFV | hemorrhage | 4 |
| 89 | 60/M | Acoustic neuromas | sigmoid sinus | CPS | no change | 6 |
| 90 | 64/F | Meningioma | temporal | SMCV | edema | 5 |
| 91 | 68/M | Meningioma | midline | PFV | hemorrhage | 4 |
| 92 | 61/F | Meningioma | suboccipital | Postcentral vein | edema | 5 |
| 93 | 39/M | Gliomas | transcortical | AFV | no change | 5 |

**GOS**: Glasgow Outcome Scale, **AFV**: Anterior frontal vein **SPV**: Superior petrosal vein, **SMCV**: Superficial middle cerebral vein, **CCBV**: Cerebellar cortical bridging vein, **MFV**: Middle frontal vein, **PFV**: Posterior frontal vein, **I/GCV**: Internal/great cerebral vein, **CSH**: chronic subdural hematoma, **CPS:** Cortical pial system
